# SIX1 Activation Is Involved in Cell Proliferation, Migration, and Anti-inflammation of Acute Ischemia/Reperfusion Injury in Mice

**DOI:** 10.3389/fmolb.2021.725319

**Published:** 2021-08-26

**Authors:** Yong Jin, Manling Zhang, Meishuang Li, Hong Zhang, Lihua Zhao, Cheng Qian, Shensen Li, Hao Zhang, Min Gao, Binbin Pan, Rongfeng Li, Xin Wan, Changchun Cao

**Affiliations:** ^1^ Department of Nephrology, Sir Run Run Hospital, Nanjing Medical University, Nanjing, China; ^2^ Jiangsu Key Laboratory of Xenotransplantation, Nanjing Medical University, Nanjing, China; ^3^ Department of Nephrology, Nanjing First Hospital, Nanjing Medical University, Nanjing, China

**Keywords:** ischemia/reperfusion, sine oculis homeobox 1, tubular epithelial cells, monocytes/macrophages, nuclear factor-κB

## Abstract

Nephrogenic proteins are re-expressed after ischemia/reperfusion (I/R) injury; however, the role of these proteins is still unknown. We found that sine oculis homeobox 1 (SIX1), a developmentally regulated homeoprotein, is reactivated in tubular epithelial cells after I/R injury associated with cell proliferation/migration and anti-inflammation. We demonstrated that SIX1 promoted cell proliferation by upregulating cyclin and glycolytic genes, and might increase cell migration by upregulating the expression of matrix metalloproteinase 9 (MMP9) directly or indirectly in the cell model. Notably, SIX1 targeted the promoters of the amino-terminal enhancer of split (*AES*) and fused in sarcoma (*FUS*), which are cofactors of nuclear factor-κB (NF-κB) subunit RELA, and then inhibited the transactivation function of RELA. The expression of monocyte chemotactic protein-1 (*MCP-1*) was decreased by the SIX1-mediated NF-κB pathway. Our results showed that the expression of cyclin, glycolytic genes, and MMP9 were significantly increased, and the infiltration of monocytes/macrophages (Mophs) was suppressed in SIX1 overexpression kidney at 1, 2, and 3 days after reperfusion. The overexpression of SIX1 resulted in reducing kidney damage from I/R injury in mice by promoting cell proliferation and migration and by inhibiting inflammation. Our study provides evidence that SIX1 involved in cell proliferation, migration, and anti-inflammation in the I/R model, which might be a potential therapeutic target that could be used to ameliorate kidney damage.

## Introduction

The clinical syndrome of acute kidney injury (AKI) is characterized by a rapid fall in the glomerular filtration rate, which frequently leads to chronic kidney disease (CKD), end-stage renal disease, and mortality (Thadhani et al., 1996; Coca et al., 2012; Chawla et al., 2014). It is well known that ischemia/reperfusion (I/R) injury can cause AKI (Bonventre and Weinberg, 2003). Tubular epithelial cells (TECs) are sensitive to acute ischemia, and a prolonged ischemic episode leads to epithelial cell death (Oberbauer et al., 1999; Kulkarni et al., 2014). Because of the kidney’s capacity to repair itself, the nephron structure and function are able to recover after prolonged ischemia (El Sabbahy and Vaidya, 2011). An increasing amount evidence has suggested that surviving TECs play an active role in cell proliferation and migration and in the response to inflammation, which contributes to the repair of the epithelium (Duffield et al., 2005; Humphreys et al., 2008).

The surviving TECs dedifferentiate, migrate along the basement membrane, and proliferate to restore cell numbers, which results in the restoration of the functional integrity of the nephron (Humphreys et al., 2008). Molecules such as PAX2 and WNT4, which are expressed in the metanephric mesenchyme during kidney development but not in the mature nephron, are abundantly expressed in TECs after I/R injury (Villanueva et al., 2006; Little and Kairath, 2017; Kumar, 2018). These markers appear within the first 24 h after injury (Villanueva et al., 2006). Previous studies also demonstrated that the nephrogenic transcription factor SOX9 was upregulated, and the descendants of SOX9^+^ cells regenerated a functional tubular epithelium after I/R and nephrotoxic insults (Liu et al., 2014; Kumar et al., 2015; Kang et al., 2016). However, whether the reactivated genes are involved in cell proliferation and migration in the early stage after I/R injury has remained unclear.

The nuclear factor-κB (NF-κB) is an inducible cellular transcription factor that regulates the expression of numerous genes involved in inflammation (Guijarro and Egido, 2001). The activation of tubular epithelial NF-κB following renal I/R injury has been documented in an animal model of AKI (Markó et al., 2016). Monocyte chemotactic protein-1 (MCP-1), a major chemotactic agent for monocytes/macrophages (Mophs) is upregulated in TECs during the early stages of inflammation (Prodjosudjadi et al., 1995; Lv et al., 2018). Sung et al. demonstrated that the activation of NF-κB is necessary for IR-induced MCP-1 expression (Sung et al., 2002). In previous studies in our laboratory, it was found that the inhibition of NF-κB activities reduced the infiltration of Mophs and decreased the expression of MCP-1 (Cao et al., 2004). Recently, Liu et al. found that sine oculis homeobox 1 (SIX1), another nephrogenic transcription factor, was reactivated in macrophages and fibroblasts and then suppressed inflammation by the targeted silencing of non-canonical NF-κB (Liu et al., 2019). Therefore, whether inflammation induced by MCP-1 mediated Mophs infiltration is regulated by the Six1-NF-κB pathway remains unknown.

SIX1 plays a pivotal role in the development of organ systems (Xu et al., 2003; Kumar, 2009). In the kidney, SIX1 and the closely related family member SIX4 are functionally redundant in the control of ureteric bud formation. The deletion of SIX1 and the combined deletion of SIX1 and SIX4 resulted in severe renal hypoplasia in mice and pigs (Kobayashi et al., 2007; Wang et al., 2019). In most adult tissue, SIX1 is little expressed; however, in some cancer tissues, the re-expression of SIX1 promoted cellular proliferation, migration and stem cell-like features by regulating cyclins and matrix metalloproteinase 9 (MMP9) (Coletta et al., 2004; Wang et al., 2012; Wu et al., 2015; Zhang et al., 2019). Recent studies indicated that SIX1 could regulate the Warburg effect and tumor growth by inducing the expression of glycolytic genes (Li et al., 2018). Fused in sarcoma (FUS, a coactivator of NF-κB subunit RELA) was also regulated by SIX1 in cancer cells (Uranishi et al., 2001; Li et al., 2018). Moreover, FUS and amino-terminal enhancer of split (AES, a nuclear corepressor) was found to interact with the same amino acid sequence of RELA (Tetsuka et al., 2000; Uranishi et al., 2001). However, whether SIX1 was reactivated after I/R injury and affected cell proliferation, migration, as well as NF-κB activities thus reduced kidney damage has not been reported.

Here, we present evidence that SIX1 was reactivated in TECs after I/R injury, which involved in cell proliferation, migration, and anti-inflammation. Our results indicated that SIX1 could drive cellular proliferation by upregulating cyclin, such as *CCNA 1*, *CCND* 1, and *C-MYC*, as well as glycolytic genes (*GLUT1*, *PGK*, and *LDHA*), and might promote cell migration by increasing the expression of *MMP9*. In this study, we identified that SIX1 inhibited the transactivation function of the NF-κB subunit RELA by regulating the corepressor gene *AES* and coactivator gene *FUS* and then suppressing the expression of *MCP-1*. The overexpression of SIX1 in mouse kidney resulted in the promotion of cell proliferation and migration and reduced the infiltration of Mophs thus attenuating kidney damage. Thus, the expression of SIX1 may provide a novel therapeutic target to alleviate I/R injury.

## Materials and Methods

### Animal Models

The study protocols were approved by the Institutional Animal Care and Use Committee at Nanjing Medical University. A unilateral I/R-induced AKI model was established as previously reported (Wan et al., 2015). Male C57BL/6 mice aged 8–10 weeks were anesthetized using an intraperitoneal injection of chloral hydrate (10%, 0.35 ml/10 g). After a flank incision, the left renal pedicle was bluntly dissected and clamped by a micro vascular clamp for 45 min. During the procedure, mice were kept well hydrated by warm sterile saline at a constant temperature (37°C). After the clamps were removed, the wounds were sutured, and the mice were allowed to recover with free access to food and water. Sham-operated mice underwent identical procedures, except the clamping of the renal pedicles was omitted. The cohorts of mice were sacrificed at 1, 2, and 3 days after surgery. For 1 day group, the ligation of right kidney was done on the same day after IRI, and the other two groups (2 and 3 days) had their ligation of right kidney 1 day before sampling. The post-ischemic kidneys and the sham-operated kidneys were harvested and stored at −80°C until they were needed for further analysis. Blood samples were obtained at the time of euthanasia. Serum creatinine was measured using enzymatic method with creatininase coupled sarcosine oxidase (Creatinine (Cr) Assay kit, C011-2-1, jiancheng Bioengineering). The level of Blood Urea Nitrogen (BUN) was determined by urease method (Urea Assay kit, C013-2-1, jiancheng Bioengineering).

An *in vivo* virus transduction to overexpress Six1 in mice kidney was performed as described elsewhere (Rocca et al., 2014). In anesthetized mice, 10 μl of an AAV cocktail (AAV-Six1 or AAV-Control, 1.0×10^12^ vector genome [vg]/ml) was directly injected into the left renal pelvis at five different sites using a glass micropipette (tip OD: 30–50 μm) with a 29 G needle, and renal pedicle was not obstructed. During injection, the position of the needle should avoid the renal vein, artery and ureter, and the depth of the needle should be about 0.5 cm. The mice were subjected to renal I/R injury 2 weeks after virus injection, and the strategy was described in Supplementary Figure S2B. The HBAAV9-CMV-mSix1-3× flag and the HBAAV9-Control vectors were purchased from HanBio Technology (Shanghai, China), and the construct of AAV-Six1 vector was shown in Supplementary Figure S2A.

### Histology, Immunofluorescence, and Immunohistochemistry

Kidney sections from paraffin-embedded tissues were prepared at 4 μm thickness and stained with hematoxylin and eosin using standard procedures for histologic evaluation. The sections were blind-reviewed by arenal pathologist and scored using a previously described semiquantitative scale designed to evaluate the degree of tubulointerstitial injury according to tubular necrosis, tubular dilatation and/or atrophy, inflammatory cell infiltration, and cellar edema (Ventura et al., 2002; Cao et al., 2004). Injury was graded on a scale from 0 to 4. Higher scores represented more severe damage (maximum score, 4): 0, normal kidney; 1, minimal damage (<5% involvement of the cortex); 2, mild damage (5–25% involvement ofthe cortex); 3, moderate damage (25–75% involvement of the cortex); and 4, severe damage (>75% involvement of the cortex).

For the immunofluorescence analysis, immunostaining was processed in 3 μm OCT-embedded sections. Nonspecific adsorption was minimized by incubating the section in 1% (g/ml) Bovine Serum Albumin (BSA; Sigma-Aldrich, St. Louis, MO)in PBS for 1 h. The samples were incubated overnight at 4°C with antibodies Six1 (1:100, 10709-1-AP, Proteintech) and F4/80 (1:100, ab6640, Abcam). sections were incubated with fluorochrome-conjugated secondary antibodies (Thermo Fisher Scientific) for 1 h at room temperature and stained with a 1:10000dilution of 4′,6-diamidino-2-phenylindole (DAPI, Sigma-Aldrich, St. Louis, MO) before the cells were mounted. The samples were viewed using a confocal imaging system (710; Carl Zeiss, Oberkochen, Germany). Image acquisition, analysis, and processing were standardized in each experiment.

Immunohistochemical staining was performed using routine protocols with the antibodies of SIX1 (1:100, 10709-1-AP, Proteintech), Ki67 (1:200, ab15580, Abcam) and MMP9 (1:200, 10375-2-AP, Proteintech). After incubation with the primary antibody at 4 °C overnight, the slides were incubated with horseradish peroxidase (HRP)-conjugated secondary antibody for 1 h at room temperature. 3,3-Diaminobenzidine tetrahydrochloride was applied to the slides for developing brown color. Counterstaining was carried out with hematoxylin. All slides contained duplicate sections, from which one served as a control for secondary antibody binding specificity. The positive areas were measured in five randomly chosen fields, and quantified blindly using a Nikon 80i camera.

### Cell Culture, Cell H/R Treatment, Cell Lines Overexpression of Mouse Six1, and *Six1* Knockout Cell Lines

Human proximal tubular epithelial cells (HK2) were used in this study. HK2 cells were immortal cells (a gift from Dr. Xiubin Liang, Nanjing Medical University, China) that were cultured in Dulbecco’s Modified Eagle Medium/F-12 (Gibco, Grand Island, NY) supplemented by a 10% fetal bovine serum (FBS) (Gibco BRL) and a 1% penicillin/streptomycin solution (Gibco, Grand Island, NY) at 37°C in 5%CO_2_. After synchronization, the cells were subjected to hypoxic conditions (1% O_2_, 5% CO_2_, 95% humidity) for 6 h, and then treated under the normal conditions (5% CO_2_, 95% humidity) for 0, 4, 8, 12, and 16 h.

Mouse *Six1* cDNA (NM-009189) was cloned into the BamH I site of pCAGDNA3. This construct is referred to as pCAGDNA3-mSix1. HK2 cells were stably transfected with pCAGDNA3 (Control) or pCAGDNA3-mSix1 using Lipofectamine 3000 (Life Technologies, Carlsbad, CA) following the manufacturer’s instructions. Stable transfectants, HK2-Six1 and Control, were selected with 200 μg/ml G418 (Gibco) in 10-cm dishes for about 10 days. Individual cell colonies were selected and cultured in 24-well plates and then transferred to 12-well plates.

*Six1* knockout mouse cell lines were generated by CRISPR/Cas9. The CRISPRs were designed using a CRISPR design web tool (http://crispr.mit.edu). The sequences of sgRNAs targeting *Six1* were: 5′-CAG​CCA​TGT​CGA​TGC​TGC​CGT​CGT​T-3′. The sgRNAs were cloned into the pX330-U6-Chimeric-BB-CBh-hSpCas9 plasmid (Addgene plasmid 42,230, Watertown, MA, USA). The resulting CRISPR/Cas9 plasmids for targeting *Six1* were confirmed by sequencing. Mouse renal tubular epithelial cells (TCMK1) (iCell Bioscience Inc., Shanghai, China) were transfected with 8 μg Six1-Cas9/sgRNA plasmids with 2 μg pCMV-tdTomato vector (Clontech, Mountain View, CA) using Lipofectamine 3000 according to the manufacturer’s protocol. After a 24 h of transfection, the selected cells were placed with 150 μg/ml G418 in 10-cm dishes for 8 days. After the G418 selection, the resistant cell clones in each experimental group were pooled and collected.

### Cell Proliferation Assay

To determine the growth rate of the Control and HK2-Six1 cells, 10,000 cells per well were plated in 24-well plates, in 500 μl of DMEM/F-12 containing 10% FBS. Cells in triplicate wells were counted daily for up to 4 days using a hemocytometer. Cell proliferation was also evaluated by the BrdU incorporation assay. Briefly, the Control and HK2-Six1 cells were cultured until 90% confluent and then treated with BrdU (30 μM) at 37°C for 1h. Single-cell suspensions (1×10^6^/tube) were fixed by 500 μl of fix buffer at 4°C overnight. The cells were then washed twice with cold wash buffer and permeabilized with Permeabilization Buffer in the dark for 2 min on ice. After treatment with DNase (300 μl) for 30 min at 37°C, the cell pellet was incubated in 200 μl BrdU antibody mix (PBS containing 5 μl of PE-BrdU antibody) (KeyGen Biotech, Nanjing, China) at 4°C for 30 min. The cells were then filtered through a 70 µm nylon cell strainer (BD Biosciences). A flow cytometry analysis (30,000 cells) was performed using a BD LSRII flow cytometer (BD Biosciences).

### Wound Healing and Cell Migration Assay

The Control and HK2-Six1 cells were plated into 6-well plates and cultured until they were 90% confluent. Cell monolayers were wounded with a sterile 1 ml pipettip and then incubated with DMEM/F-12 containing 1% FBS for 24 and 48 h in an atmosphere of 5% CO_2_ at 37°C. Representative areas of the wounded monolayers containing wounds of the same width were marked and photographed. After incubation, the same areas were photographed. The extent of wound repair was evaluated by measuring the wound area using ImageJ (NIH). Each experiment was performed in triplicate wells and repeated three times.

The migration ability of the Control and HK2-Six1 cells was measured using transwell chambers (24-well plate) (Corning, Cambridge, MA) with polycarbonate membranes (8.0 μm pore size) coated with 100 μl of the extracellular matrix (ECM; Sigma-Aldrich, St. Louis, MO) on the top side of the membrane. The upper surface of the matrix was challenged with 20,000 cells, which were kept in basal medium containing 0.1% BSA (Sigma-Aldrich, St. Louis, MO). The lower chamber contained the complete medium. After 24 h and 48 h, the cells were fixed and stained. The cells and ECM on the upper surface of the membrane were carefully removed using a cotton swab. Cells that migrated through the membrane were fixed, stained with 1% crystal violet, and counted in five randomly chosen areas. The migration of cells was measured by directly counting the number of cells that migrated to the lower chamber. Each experiment was performed in triplicate wells and repeated three times.

### Quantitative Real-Time PCR and Western Blot Analysis

Total RNA was isolated from the cells and frozen tissues using TRIzol (total RNA extraction reagent; Life Technologies Corporation, Carlsbad, CA). Reverse transcription was performed with 1 μg of total RNA using HiScript II One Step RT-PCR Kit (Vazyme Biotech, Nanjing, China), and quantitative real-time PCR was performed using ChamQ™ SYBR qPCR Master Mix (Vazyme Biotech). The sequence of the primers used in the quantitative real-time polymerase chain reaction (qRT-PCR) is shown in Supplementary Table S1. *β-Actin* was used as an internal control. All samples were assayed in triplicate.

Western blotting was performed as previously described (Wang et al., 2019). Whole cells and tissues lysates were prepared in RIPA buffer (Beyotime Biotechnology, Shanghai, China). Antibodies against the following proteins were used: SIX1 (1:1000, sc-514441, Santa Cruz Biotechnology, Dallas, TX); SIX1 (1:1000, D4A8K, Cell Signaling Technology); C-MYC (1:1000, D3N8F, Cell Signaling Technology); Cyclin A1 (1:1000, ab185619, Abcam); Cyclin D1 (1:1000, ab134175, Abcam); AES (1:1000, ab137060, Abcam); FUS (1:1000, ab124923, Abcam); GLUT1 (1:1000, 21829-1-AP, Proteintech); PGK1 (1:1000, 17811-1-AP, Proteintech); LDHA (1:1000, 19987-1-AP, Proteintech); MMP9 (1:1000, 10375-2-AP, Proteintech); GAPDH (1:3000, HRP-60004, Proteintech) was used as a loading control.

### Luciferase Reporter Assay

1×10^5^ HK2-Six1 and HK2-control (Control) cells, 1×10^5^ TCMK1-Six1^−/−^ and TCMK1 wild-type (Control) cells were seeded into each well of 6-well plates. The indicated plasmids were transfected into cells with the firefly luciferase reporter of NF-κB and the renilla luciferase reporter (pNFκB-TA-luc, pRL-SV40-N, Beyotime Biotechnology) using Lipofectamine 3000 (Life Technologies). Forthe cytokine treatment, after 24 h of transfection, the cells were treated with 25 ng/ml TNFα (Novoprotein Scientific, Shanghai, China) for 24 h. Luciferase activity was measured according to the manufacturer’s protocol (Dual Luciferase Reported Gene Assay Kit, Beyotime Biotechnology). The luminescence units were measured using multimode microplate readers (Varioskan LUX, Thermo Scientific). The relative luciferase activity was quantified by adjusting it to the renilla luciferase activity and normalizing it to the experimental control.

### Chromatin Immunoprecipitation (ChIP)

The ChIP assay was performed according to the manufacturer’s instructions (Simple ChIP Plus Enzymatic Chromatin IP Kit, 9003, Cell Signaling Technology). In brief, 1.2×10^7^ cells were cross-linked with 1% formaldehyde for 10 min at room temperature and glycine was used to quench crosslinking. The cells were then washed twice with chilled PBS, and lysed in lysis buffer. The nuclei were then pelleted down by spinning at 3,000 rpm. for 5 min. Nuclei lysates were sonicated for three cycles at 20 s. on and 30 s off to yield fragments from 150 to 900 bp using Bioruptor (SONICS and MATERIALS). Nuclei lysates were then immunoprecipitated by ChIP grade antibodies against SIX1 (D4A8K, Cell Signaling Technology) or a normal IgG using protein G agarose beads. Then the DNA and transcriptional factor complexes were uncross-linked to obtain a pure DNA fragment. A quantitative PCR was then performed using the following primers: *AES* promoter (forward 5′-CTT CAG GTC GCT TTA GGG GTG GAG G-3’; reverse 5′-GGC AGG TCA GAT GGT GGG GAC AGT-3′) and *FUS* promoter (forward 5′-CAC AGG AAT CTC GGT TCC ACC C-3’; reverse 5′-CAG GAC TAG CCC ACG ATC TAT CTC C-3′) in human cells. Fold enrichment was normalized to the experimental IgG control.

### Statistical Analysis

The data were expressed as mean ± standard deviations (SD). A statistical analysis was performed using the student *t*-test when only two value sets were compared. A one-way ANOVA followed by Dunnett’s test or Bonferroni correction was conducted when the data involved three or more groups. The differences were evaluated using SPSS10.0 software (SPSS, Chicago, IL, USA). *p* < 0.05 was considered statistically significant.

## Results

### Six1 Reactivation in TECs After I/R Injury

**FIGURE 1:**
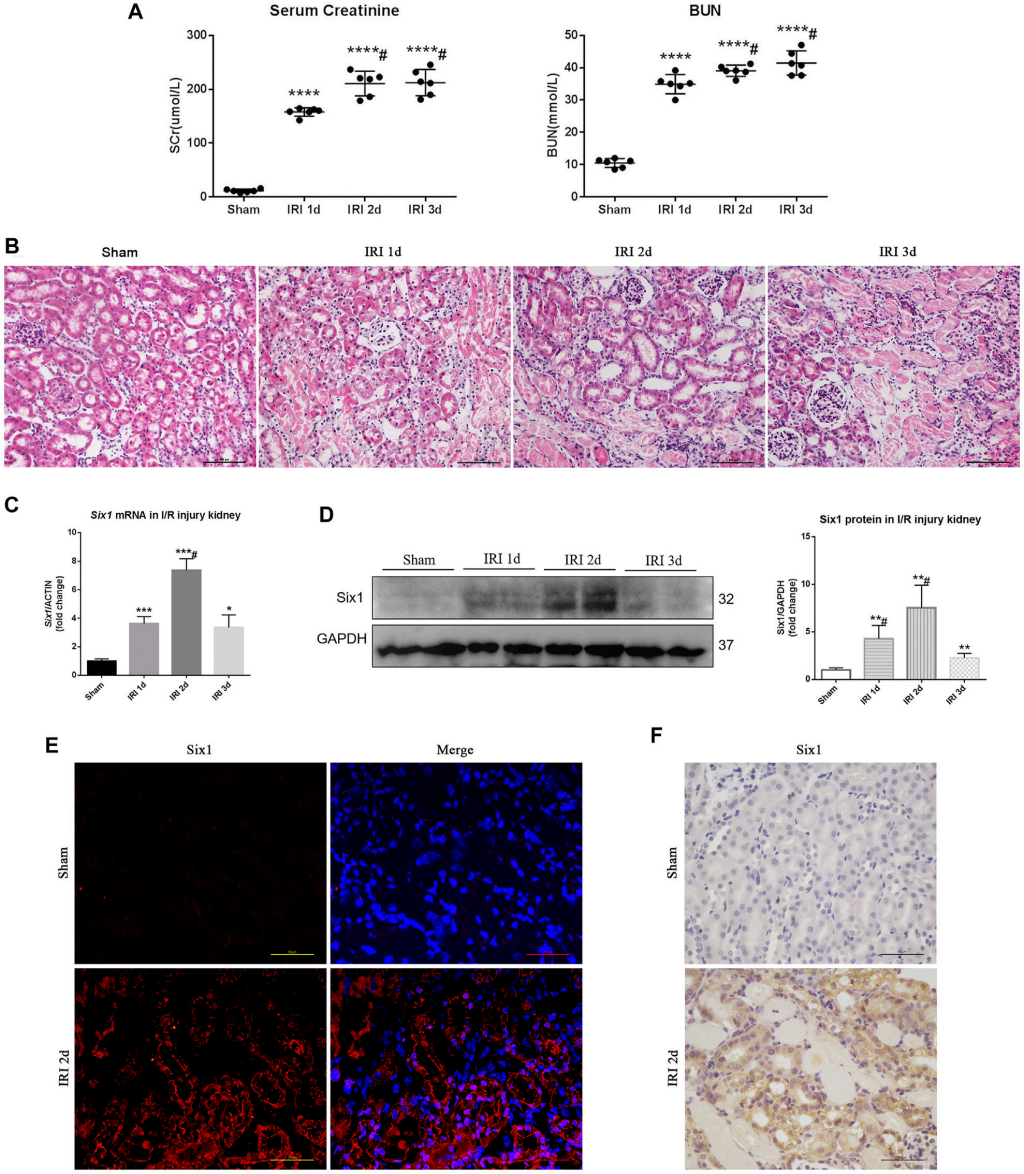
Six1 is upregulated in the proximal tubular epithelial cells (TECs) after unilateral ischemia/reperfusion injury (IRI). **(A)** Serum creatinine (SCr) and blood urea nitrogen (BUN) levels in I/R injury. **(B)** Histological changes (H and E staining). Bar = 100 μm. **(C)** mRNA expression of *Six1* in the I/R injured kidney was assessed by qRT-PCR. **(D)** Western blotting analysis of Six1 expression in the I/R injured kidney. **(E)** Immunofluorescence analysis of Six1 in IRI 2 days kidneys or sham. Bar = 50 μm. **(F)** Immunohistochemistry analysis of Six1 in IRI 2 days kidneys or sham. Bar = 50 μm. Data are mean ± SD for groups of six mice. *p<0.05, **p<0.01, ***p<0.001, ****p<0.0001 versus sham (*t*-test); #*p*<0.05 versus IRI 2 days (Bonferroni correction, two comparisons were made). IRI 1d, 1 day after IRI; IRI 2d, 2 days after IRI; IRI 3d, 3 days after IRI; GAPDH, glyceraldehydes-3-phosphate dehydrogenase.

An I/R-induced AKI model was established as previously reported (Wan et al., 2015). After I/R injury, mice were weak but still alive, and were sacrificed at three time points: 1 day (1d), 2 days (2d), and 3 days (3d). Serum creatinine and BUN were significantly increased in the I/R injury (IRI) mice at 1, 2, and 3 days (Figure 1A). Histologically, TEC injury and protein casts were found in kidneys at 1, 2, and 3 days (Figure 1B). The mRNA expression of *Six1* was significantly increased in the injured kidney at 1 day (3.6-fold), and peaked at 2 days (7.3-fold), which preceded a significant decrease at 3 days (3.3-fold), but all increased significantly compared with the sham-operated kidney (Figure 1C). Six1 protein expression was also detected by Western blotting (Figure 1D). To determine which cells expressed Six1 in the injured kidney, we performed an immunofluorescence and immunohistochemistry analysis on kidney sections. In the IRI 2 days kidney, Six1^+^ cells were present in TECs, whereas the expression of Six1 was undetectable in the sham-operated kidney (Figures 1E,F).

### I/R Induced Cell Proliferation/Migration and Inflammatory Response

**FIGURE 2:**
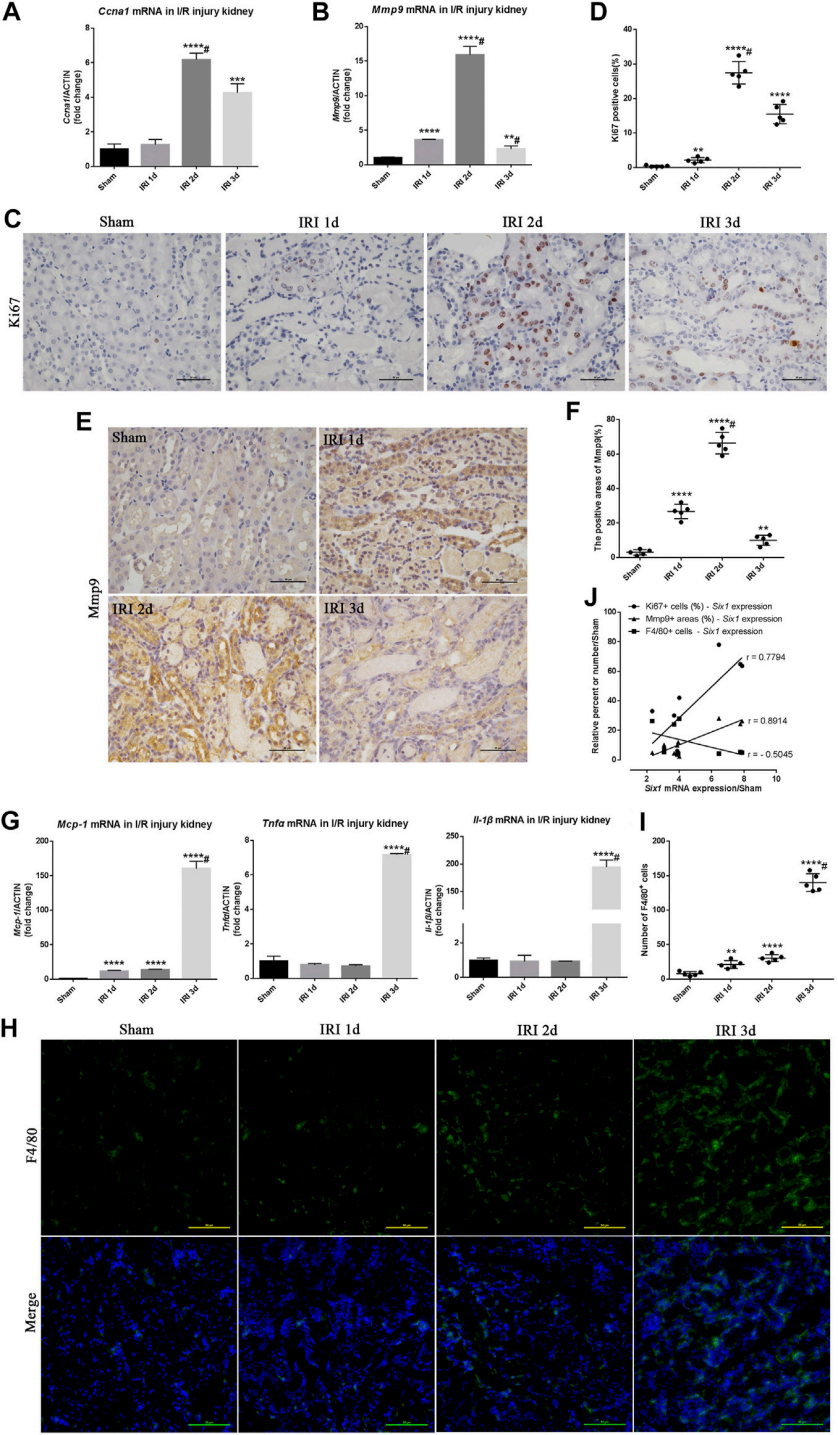
Cell proliferation/migration and inflammatory response during unilateral ischemia/reperfusion injury (IRI). **(A, B)** mRNA expression of *Ccna 1* and matrix metalloproteinase 9 (*Mmp9*) in the IRI kidney assessed by qRT-PCR. **(C, E)** Immunohistochemistry analysis for Ki67 (a marker of cell proliferation) and Mmp9 in IRI kidneys or sham. Bar = 50 μm. **(D, F)** Scatter diagram presentation of the percent of the Ki67 positive cells and Mmp9 positive areas at different time points after IRI. #*p*<0.05 versus IRI 2 days (Bonferroni correction; two comparisons were made).**(G)** qRT-PCR analysis of inflammatory factors (*Mcp-1*, *Tnfα*, and *Il-1β*) in the kidney. **(H)** Representative F4/80 stained kidney sections from IRI or sham mice. Bar = 50 μm.**(I)** Scatter diagram presentation of the numbers of the F4/80 positive cells at different time points after IRI. #*p*<0.05 versus IRI 3 days (Bonferroni correction; two comparisons were made). **(J)** The correlation statistical analysis between *Six1* mRNA expression and related molecular markers (Ki67, *p* = 0.0133; Mmp9, *p* = 0.0012; F4/80, *p* = 0.166) expression in IRI kidneys. Data are mean ± SD for groups of six mice. **p<0.01, ***p<0.001, ****p<0.0001 versus sham (*t*-test). IRI 1d, 1 day after IRI; IRI 2d, 2 days after IRI; IRI 3d, 3 days after IRI.

To investigate cell proliferation and migration after I/R injury, we compared *Ccna 1* and *Mmp9* mRNA expression in the injured kidney with the sham-operated kidney. The expression of *Ccna 1* and *Mmp9* was significantly elevated (6.1-fold and 15.9-fold, respectively) in the IRI 2 days kidney (Figures 2A,B). Immunohistochemistry analysis further revealed that Ki67 and Mmp9 were expressed in TECs at 1, 2ays, and 3 days after I/R injury (Figures 2C,E). Concomitantly, the percent of Ki67^+^ TECs and Mmp9 positive areas was significantly increased in the injured kidney at 2 days (27.5 and 65%, respectively) and positively correlated with the expression of *Six1* in the injured kidney at 1, 2, and 3 days (r = 0.7794, *p* = 0.0133 and r = 0.8914, *p* = 0.0012, respectively) (Figures 2D,F,J).

The effects of I/R on *Mcp-1* and inflammation genes (*Tnfα* and *Il-1β*) mRNA expression were examined using quantitative real-time PCR. As shown in Figure 2G, there was a significant elevation of *Mcp-1*, *Tnfα*, and *Il-1β* mRNA levels (160-fold, 7.1-fold, and 194-fold, respectively) in the IRI 3 days kidney compared with the sham-operated kidney. The immunofluorescence analysis further revealed that more F4/80^+^ Mophs were present in the IRI 3 days kidney (Figures 2H,I). Although there was no significant negatively correlated between the number of F4/80^+^ Mophs and the expression of *Six1* in the injured kidney (r = –0.5045, *p* = 0.166), *Six1* expression could obviously reduce the number of infiltrated F4/80^+^ Mophs (Figure 2J). Moreover, stronger expression of Six1 was observed in the IRI 2 days kidney, whereas there was little Mophs infiltration. These results implied that the TECs proliferation/migration and Mophs infiltration was associated with the time-course expression of tubular Six1 in the I/R injury kidney.

### Reactivation of Human SIX1 was Induced by Hypoxia/Reoxygenation, and Overexpression of Mouse *Six1* Promoted Cell Proliferation by Upregulating Cyclin and Glycolytic Genes

**FIGURE 3:**
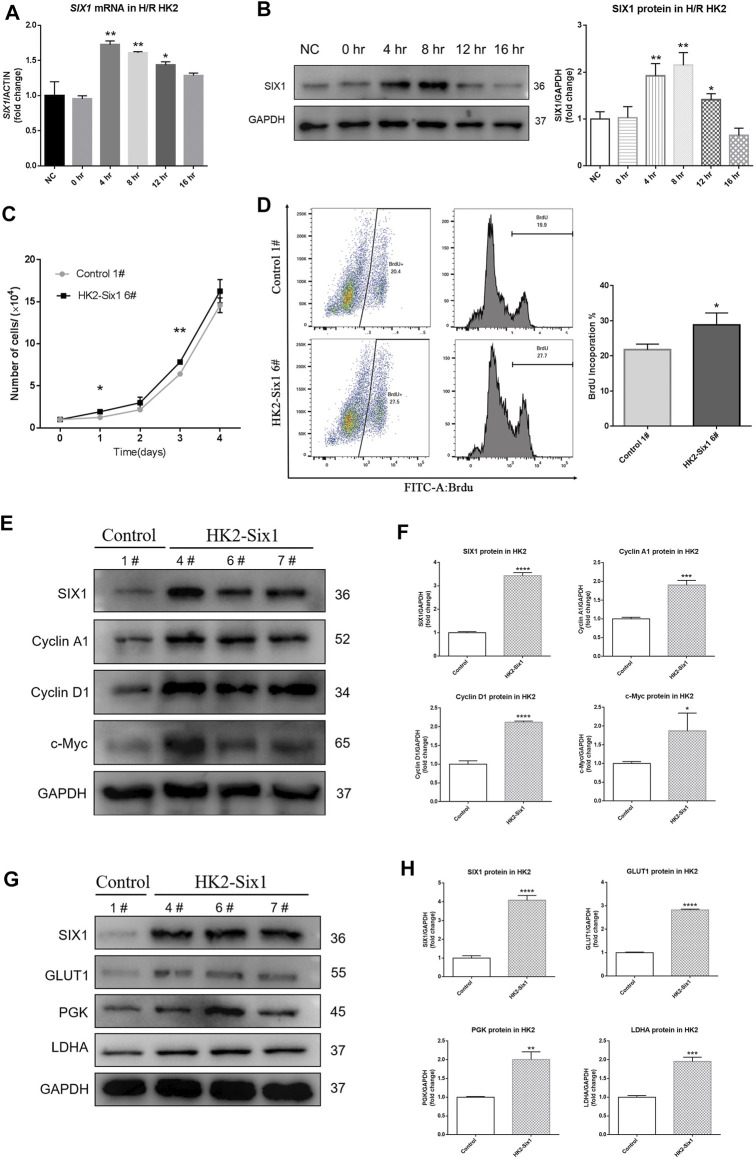
Hypoxia/reoxygenation (H/R) induces human SIX1 expression, and the overexpression of mouse Six1 promotes cell proliferation. **(A, B)** mRNA and protein expression of *SIX1* in H/R treated HK2 cells assessed by qRT-PCR and Western blotting. **(C)** Cell proliferation was determined by directly counting cells at the days indicated in the mouse Six1 overexpression HK2 cell line (HK2-Six1 6#) and the Control 1#. **(D)** Analysis by flow cytometry of BrdU-labeled cells in the HK2-Six1 6# and Control 1# cell lines. **(E, F)** Western blotting for SIX1 and cyclins (Cyclin A1, Cyclin D1, and c-Myc) in HK2-Six1 cell lines (4#, 6# and 7#) and Control 1# cell line. **(G, H)** SIX1 and glycolytic molecules (GLUT1, PGK, and LDHA) in HK2-Six1 cell lines (4#, 6# and 7#) and the Control 1# cell line assessed by Western blotting. Data are expressed as mean ± SD. *p<0.05, **p<0.01, ***p<0.001, ****p<0.0001 versus the NC or control group (*t*-test). NC, normal conditions; 0 h, 0 h after H/R; 4 h, 4 h after H/R; 8 h, 8 h after H/R; 12 h, 12 h after H/R; 16 h, 16 h after H/R; GAPDH, glyceraldehydes-3-phosphate dehydrogenase.

To determine whether hypoxia/reoxygenation (H/R) affected the expression of SIX1, we measured the mRNA and protein levels of SIX1 in human proximal epithelial tubule cells (HK2) subjected to H/R. This protocol reproduced *in vitro* the stimuli and effects of renal I/R *in vivo* (Sáenz-Morales et al., 2006). The expression of *SIX1* mRNA was significantly increased in the HK2 cells at 4–12 h of reoxygenation before decreasing to a low level at 16 h as compared with the HK2 cells under normal conditions (NC) (Figure 3A). A similar pattern of protein expression was observed by Western blotting (Figure 3B).

Initially, we examined mouse Six1 overexpression to determine how it affected the proliferation of HK2 cells by counting them. As shown in growth curves (Figure 3C), there were higher cell numbers in the Six1 overexpression cell line (HK2-Six1) (Supplementary Figure S1A–C) groups at different time points compared to the control cell line (Control). We next assessed the effects of Six1 on cell cycle progression by 5-bromodeoxyuridine (BrdU) uptake. After the cells were treated with BrdU, the number of BrdU-labeled cells was significantly higher in HK2-Six1 than in the Control (28.8 vs. 21.7%) (Figure 3D). The Western blot analysis further revealed that the protein levels of human Cyclin A1, Cyclin D1, and c-Myc were dramatically increased in the HK2-Six1 cells (Figures 3E,F). Moreover, the transfection of mouse *Six1* stimulated the protein expression of human glycolytic genes (*GLUT1*, *PGK*, and *LDHA*) (Figures 3G,H). These results demonstrated that the overexpression of mouse *Six1* promoted cell proliferation by elevating cell cycle progression and energy metabolism.

### Cell Migration Enhanced by Mouse *Six1* Expression

**FIGURE 4:**
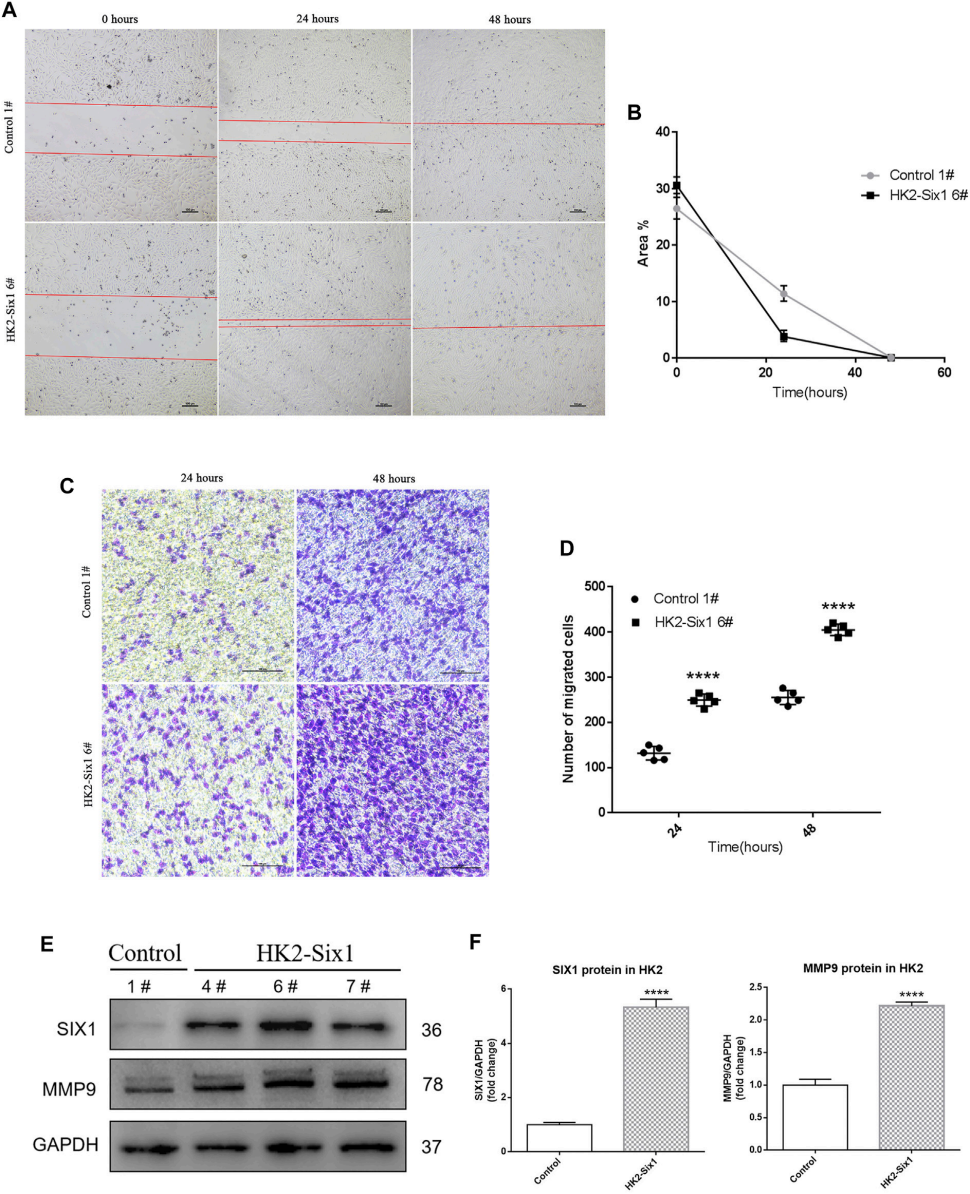
| Mouse Six1 might promote migration by upregulating matrix metalloproteinase 9 (MMP9) expression in HK2 cells in vitro. (A, B) Cell migration was determined in the mouse Six1 overexpression HK2 cell line (HK2-Six1 6#) and the Control 1# cell line using a wound healing assay at 0 h, 24 h, and 48 h. Bar = 100 μm. The area of the wound was quantified to determine the extent of wound repair. (C, D) Extracellular matrix (ECM) coated transwell assay analysis cell migration in HK2-Six1 6# and Control 1# cell lines at 24 and 48 h Bar = 100 μm. The number of cells in the lower chamber was quantified after crystal violet staining. (E, F) Protein expression of SIX1 and MMP9 in HK2-Six1 cell lines (4#, 6# and 7#) and the Control 1# cell line was assessed by Western blotting. Data are expressed as mean ± SD. ****p<0.0001 versus the control group (t-test). GAPDH, glyceraldehydes-3-phosphate dehydrogenase.

Because Mmp9 expression was strongly associated with the time-course expression of Six1 *in vivo*, we sought to identify changes in cell migration involved with the overexpression of mouse *Six1*. In the wound healing assay, HK2-Six1 exhibited enhanced cell migration compared with the Control (Figures 4A,B). We next conducted a cell migration assay for 24 h and 48 h using transwells. Based on our observations, the number of migrated cells was significantly increased in HK2-Six1 at 24 h and 48 h compared with the control cells (Figures 4C,D). To fully explore the underlying mechanism by which Six1 overexpression in HK2 cells affects its migration, we examined the human *MMP-9* protein expression level in HK2-Six1 cells. A higher level of expression of MMP-9 was found in HK2-Six1 cells compared to the control cells (Figures 4E,F). These findings suggest that Six1 might promote cell migration by increasing the expression of MMP9 directly or indirectly.

### Six1 Inhibited NF-κB Activities and *MCP-1* Expression

**FIGURE 5:**
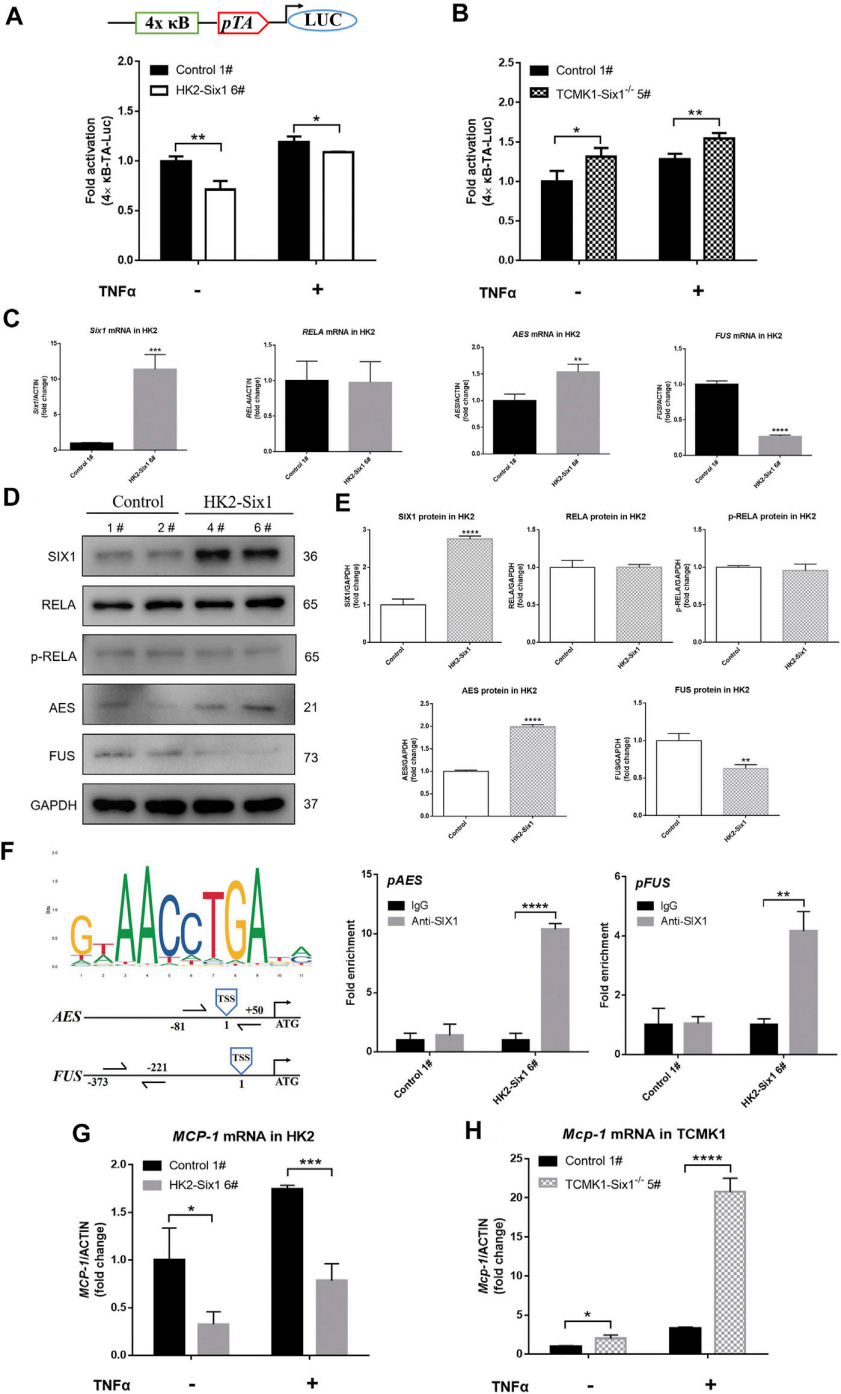
Mouse Six1 suppresses monocyte chemotactic protein-1 (*MCP-1*) expression by inhibiting NF-κB activation. **(A)** pNFκB-TA-luc reporter containing four NFκB binding sites (top). pNFκB-TA-luc co-transfected with pRL-SV40-N (internal control reporter plasmid) in mouse Six1 overexpression HK2 cells (HK2-Six1 6#) and the control 1#. Cells were harvested 24 h after transfection or treatment with 25 ng/ml TNFα for another 24 h, and luciferase activity was measured (bottom). **(B)** Plasmids (pNFκB-TA-luc and pRL-SV40-N) were co-transfected into the mouse renal tubular epithelial cells *Six1* knockout (TCMK1-Six1^−/−^ 5#) and the Control 1#. Luciferase activity was measured. The relative luciferase activity was quantified by adjusting it to the renilla luciferase activity and untreated control samples were normalized to 1.0. **(C)** mRNA expression of mouse *Six1*, human NFκB subunit *RELA*, human cofactors genes of RELA (*AES* and *FUS*) were assessed by qRT-PCR in the HK2-Six1 6# and the Control 1# cell lines. **(D, E)** Western blotting was applied to SIX1, RELA, *p*-RELA, AES, and FUS in HK2-Six1 cell lines (4# and 6#) and the Control cell lines (1# and 2#). **(F)**SIX1 DNA binding compatible motif (ACCTGA) in *AES* and *FUS* gene promoter regions and chromatin immunoprecipitation (ChIP) analysis of SIX1 occupancy of the *AES* and *FUS* genes from HK2-Six1 6# and the control 1# cell lines. The location of each primer set compared to the transcription start site (TSS) is shown (left). IgG control samples were normalized to 1.0. **(G, H)** After treatment by 25 ng/ml TNFα for 24 h or no treatment, mRNA expression of human *MCP-1* and mouse *Mcp-1* assessed by qRT-PCR in HK2-Six1 6# and Control 1#, TCMK1-Six1^−/−^ 5# and Control 1#, respectively. Data are expressed as mean ± SD. *p<0.05, **p<0.01, ***p<0.001, ****p<0.0001 versus the Control group (*t*-test). GAPDH, glyceraldehydes-3-phosphate dehydrogenase.

It is well known that NF-κB is activated in kidney during I/R, which is regulated by several factors, including SIX proteins (Marko et al., 2016; Liu et al., 2019). To determine whether mouse Six1 affects the activation of NF-κB in TECs, we analyzed luciferase reporter expression driven by four NF-κB binding sites (4 × κB) (Figure 5A [top]). The stable transfection of HK2 cells with mouse *Six1* inhibited the basal activities of NF-κB and the TNFα-stimulated activation of NF-κB, respectively (Figure 5A [bottom]). In addition, mouse renal TEC *Six1* knockout cell lines (TCMK1-Six1^−/−^) (Supplementary Figure S1D,E) enhanced the stimulation of 4 × κB-LUC under both quiescent and stimulated conditions (Figure 5B). These results suggest that Six1 represses the basal and TNFα-stimulated activities of NF-κB.

To reveal the mechanism of the inhibition of NF-κB by Six1, we examined the expression and activation of human *RELA* (*p65*), a member of NF-κB family responsible for transcriptional activation (Schmitz et al., 1995). There was no significant difference in the RNA and protein level of *RELA* as well as phosphorylated-RELA (*p*-RELA) level between the HK2-Six1 cells and the control cells (Figures 5C–E). Previous studies indicated that specific protein-protein interactions with RELA determine its transcriptional competence (Tetsuka et al., 2000; Uranishi et al., 2001). We next examined the RNA expression of corepressors and coactivators of RELA. The results showed that human amino-terminal enhancer of split (*AES*, a corepressor gene of RELA) RNA expression was markedly elevation, but fused in sarcoma (*FUS*, a coactivator gene of RELA) RNA expression was suppressed dramatically in the HK2-Six1 cells (Figure 5C). A Western blot analysis further confirmed that the pattern of protein expression of *AES* and *FUS* was in keeping with RNA expression in HK2-Six1 cells (Figures 5D,E). As SIX1 DNA binding motifs has been defined for human, we searched and obtained SIX1 DNA binding motifs for human on the website of JASPAR, and found that SIX1 DNA binding compatible motif (ACCTGA) is in *AES* and *FUS* gene promoter regions by sequence alignments (counting position from the gene transcription start sites, the location of compatible motif was -1008 in *AES* and -2948 in *FUS* respectively) (Figure 5F). The chromatin immunoprecipitation (ChIP) analysis showed that SIX1 protein bound the promoter regions of the *AES* and *FUS* genes, indicating that it might be primed for transcriptional activation and inhibition, respectively (Figure 5F). These results showed that mouse Six1 inhibited the transactivation function of the RELA subunit of NF-κB by regulating the expression of corepressor gene *AES* and coactivator gene *FUS*.

Our previous laboratory studies clearly demonstrated that enhanced MCP-1 expression was responsible for the activation of NF-κB (Cao et al., 2004). To clarify whether *MCP-1* expression was regulated by the SIX1-mediated NF-κB pathway, we examined human *MCP-1* and mouse *Mcp-1* RNA expression in mouse Six1 overexpression and *Six1* knockout cell lines under both quiescent and TNFα-stimulated conditions, respectively. As shown in Figure 5G, the human *MCP-1* mRNA level was more attenuated in the HK2-Six1 cells compared with the control cells. However, mouse *Mcp-1* was significantly increased in TCMK1-Six1^−/−^ (Figure 5H). Thus, mouse Six1 repressed the basal and TNFα-stimulated expression of the chemokine MCP-1 in TECs.

### Overexpression of Six1 in the Mouse Kidney Attenuated IR Injury

**FIGURE 6:**
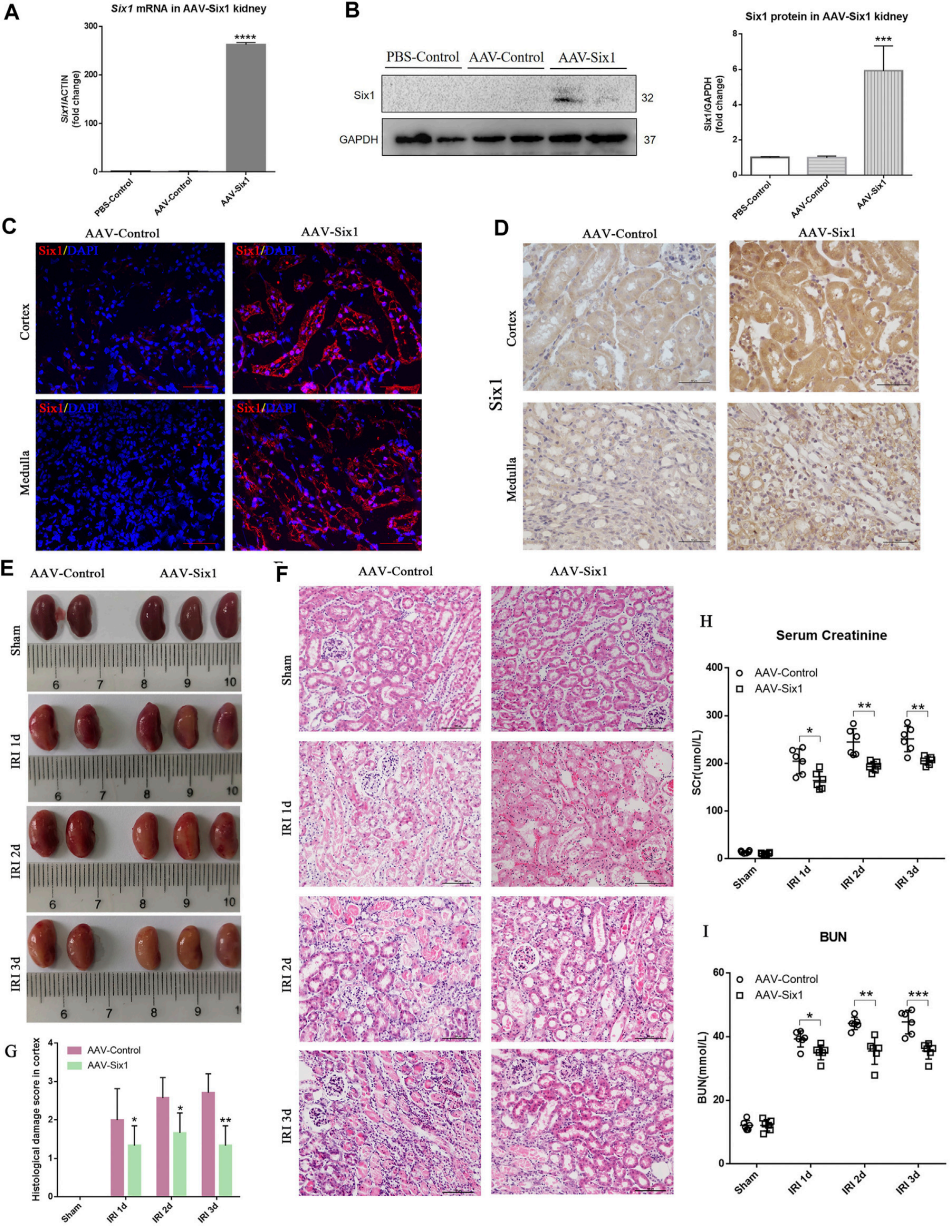
Overexpression of Six1 in mouse kidney before unilateral ischemia/reperfusion injury (IRI) attenuates renal damage. **(A, B)** mRNA and protein expression of *Six1* in adeno-associated viral vector serotype 9 (AAV9)-Six1 treated (AAV-Six1), phosphate buffer saline treated (PBS-Control) and AAV empty vector treated (AAV-Control) kidney assessed by qRT-PCR and Western blotting. **(C)** Immunofluorescence analysis of Six1 in AAV-Six1 and the AAV-Control group before IRI. Bar = 50 μm. **(D)** Immunohistochemistry analysis of Six1 in AAV-Six1 and the AAV-Control group before IRI. Bar = 50 μm. **(E)** Images of kidneys at 1, 2, and 3 days after IRI or kidneys sham-operated in AAV-Six1 and the AAV-Control group. **(F, G)** Representative images of H and E staining of the renal cortex at the indicated time in AAV-Six1, AAV-Control group, and the histological damage score. Bar = 100 μm. **(H, I)** Serum creatinine (SCr) and blood urea nitrogen (BUN) levels in AAV-Six1 and AAV-Control mice after IRI. Data are mean ± SD for groups of six mice. *p<0.05, **p<0.01, ***p<0.001, ****p<0.0001 versus AAV-Control (*t*-test). #*p*<0.05 versus IRI 1 day in AAV-Control (Bonferroni correction, two comparisons were made). IRI 1d, 1 day after IRI; IRI 2d, 2 days after IRI; IRI 3d, 3 days after IRI; GAPDH, glyceraldehydes-3-phosphate dehydrogenase.

To study the effects of Six1 overexpression on I/R injury, we injected the adeno-associated viral vector serotype 9 (AAV9)-Six1into an uninjured renal pelvis at 14 days before I/Rinjury. The RNA and protein expression of Six1 were both increased in the AAV-Six1 kidney compared with the phosphate buffer saline (PBS-Control) and the AAV empty vector control kidney (AAV-Control) (Figures 6A,B). Immunofluorescence and immunohistochemistry analysis of kidney sections revealed some Six1^+^ cells were present in cortex and medulla TECs in AAV-Six1 group, but the expression of Six1 was undetectable in AAV-Control group (Figures 6C,D). Animals that underwent 45 min of unilateral renal ischemia and reperfusion at 1, 2, and 3 days exhibited a significant degree of renal damage, which was evident in renal edema, compared with the sham-operated kidney. Compared with the AAV-Control group, the AAV-Six1 infected group exhibited remission of renal edema (Figure 6E). As expected, the histological analysis of kidney sections at 1, 2, and 3 days after unilateral I/R injury revealed significant tubulointerstitial damage, including tubular atrophy, flattening and sloughing of TECs, protein cast formation, and inflammatory cell infiltration, all of which were markedly ameliorated in the AAV-Six1 group (Figures 6F,G).

Notably, compared with AAV-Control, the administration of AAV-Six1 resulted in a significantly lower level of serum creatinine at 1 day (168.5 ± 8.918 vs. 204.4 ± 10.70, n = 6), 2 days (193.4 ± 4.297 vs. 244.6 ± 11.56, n = 6), and 3 days (205.2 ± 3.603 vs. 251.1 ± 10.99, n = 6) after I/Rinjury (Figure 6H). The serum creatinine levels were decreased 17.6% at IRI 1 day, 20.9% at IRI 2 days and 18.3% at IRI 3 days after Six1 overexpression in the animal model. In the AAV-Control group, the level of serum creatinine was significantly increased at IRI 3 days compared with IRI 1 day after analysis by Bonferroni correction. Meanwhile, the level of BUN was also significantly decreased in AAV-Six1 group compared to AAV-Control group at 1 day (35.02 ± 0.9528 vs 39.25 ± 1.037, n = 6), 2 days (35.49 ± 1.719 vs 44.15 ± 0.7915, n = 6), and 3 days (35.51 ± 1.045 vs 44.58 ± 1.518, n = 6) (Figure 6I). In the AAV-Control group, the levels of BUN were significantly increased at IRI 2d and 3 days compared with IRI 1 day after analysis by Bonferroni correction. These results suggested that Six1 overexpression in mouse kidney reduced the damage caused by I/R at 1, 2, and 3 days.

### Six1 Promoted Cell Proliferation/Migration and Suppressed Mophs Infiltration *in vivo*

**FIGURE 7:**
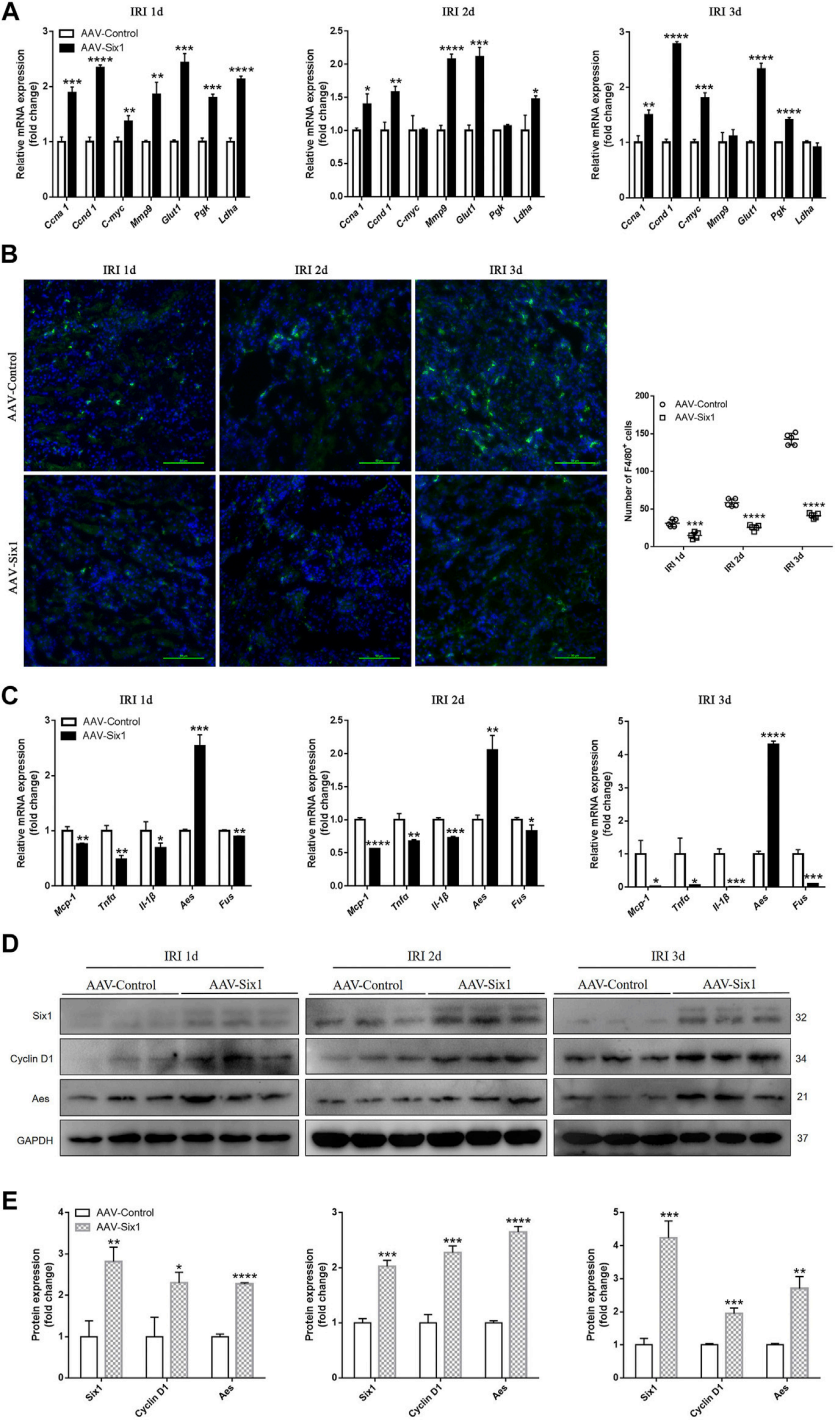
Six1 promotes cell proliferation/migration and suppresses monocytes/macrophages (Mophs) infiltration *in vivo*. **(A)** mRNA expression of cyclin genes (*Ccna 1*, *Ccnd 1* and *C-myc*), glycolytic genes (*Glut1*, *Pgk* and *Ldha*) and *Mmp9* in adeno-associated viral vector serotype 9 (AAV9)-Six1 treated (AAV-Six1), and AAV empty-vector treated (AAV-Control) kidneys at 1, 2, and 3 days after ischemia/reperfusion injury (IRI) assessed by qRT-PCR. **(B)** Representative F4/80 stained kidney sections from AAV-Six1 and AAV-Control mice at indicated time after IRI and quantification of the number of F4/80^+^ Mophs. F4/80 (green) and 4′,6-diamidino-2-phenylindole (DAPI, blue). Bar = 50 μm. **(C)** qRT-PCR analysis of inflammatory factors (*Mcp-1*, *Tnfα*, *Il-1β*), and cofactor genes of NFκB subunit RELA (*Aes* and *Fus*) in AAV-Six1 and AAV-Control IRI kidneys. **(D, E)** Protein expression of Six1, Cyclin D1 and Aes in AAV-Six1 and AAV-Control kidneys at 1, 2, and 3 days after IRI were assessed by Western blotting. Data are mean ± SD for groups of three mice. *p<0.05, **p<0.01, ***p<0.001, ****p<0.0001 versus AAV-Control (*t*-test). IRI 1d, 1 day after IRI; IRI 2d, 2 days after IRI; IRI 3d, 3 days after IRI; GAPDH, glyceraldehydes-3-phosphate dehydrogenase.

Based on our findings in the cell models, we analyzed cell proliferation and migration to determine whether they were increased by upregulating mouse cyclins, glycolytic genes, and *Mmp9* in the AAV-Six1 kidney. As shown in Figure 7A, the RNA expression levels of cyclins (*Ccna 1*, *Ccnd 1*, and *C-myc*), glycolytic genes (*Glut1*, *Pgk*, and *Ldha*), and *Mmp9* were increased in the AAV-Six1 kidney compared to the AAV-Control kidney after I/R injury. The infiltration of Mophs was assessed by immunostaining analysis. Compared with the AAV-Control group, the number of interstitial cells demonstrating F4/80 staining of Mophs was significantly decreased at 1, 2, and 3 days after I/R injury in the AAV-Six1 group (Figure 7B). We also found that the RNA expression of genes (*Mcp-1*, *Tnfα*, and *Il-1β*) correlated with Mophs infiltration was significantly attenuated in the AAV-Six1 kidney. Moreover, mouse *Aes* RNA expression was promoted and *Fus* RNA expression was suppressed at 1, 2, and 3 days after I/R injury in the AAV-Six1 kidney (Figure 7C). A Western blot analysis further confirmed that the protein expression levels of Cyclin D1 and Aes were positively correlated with Six1 overexpression in IRI 1 day, IRI 2 days, and IRI 3 days kidney (Figures 7D,E). In addition, the RNA expression of genes (*Ccna 1*, *Ccnd 1*, *C-myc*, *Mmp9*, *Glut1* and *Aes*) were significantly increased in the AAV-Six1 kidney compared with the AAV-Control kidney before I/R injury (Supplementary Figure S2C). Thus, these results indicated that Six1 overexpression promoted cell proliferation and migration by upregulating cyclins, glycolytic genes, and *Mmp9* and suppressed Mophs infiltration by repressing the expression of *Mcp-1* in IRI kidney.

## Discussion

**FIGURE 8:**
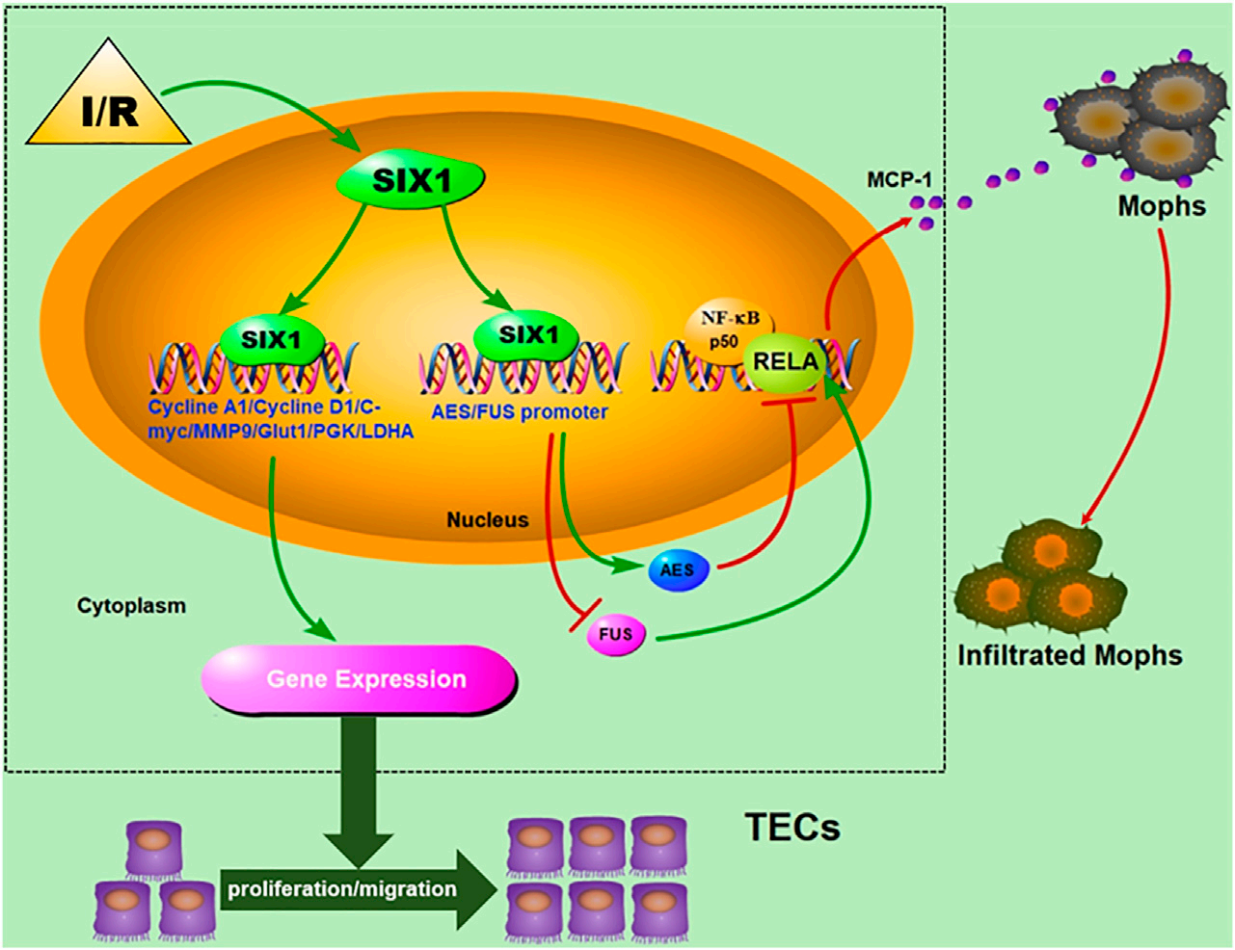
Schematic depicting the effects of Six1 activation in renal tubular epithelial cells (TECs) on cell proliferation/migration and anti-inflammation and underlying mechanism.

Previous observations of the reactivation of key developmental gene (s) after AKI have suggested that activated genes may play a crucial role during recovery from kidney injury (Kumar et al., 2014). However, whether the reactivated gene was involved in TEC restoration and inflammatory response in the early phase of I/R injury is unclear. In the present study, we demonstrated that Six1 was upregulated in the proximal tubules, and its expression was closely associated with cell proliferation and migration and tubulointerstitial inflammation after an I/Rinjury. Our findings *in vitro* suggest that the expression of human SIX1 is induced by hypoxia/reoxygenation, and the overexpression of mouse Six1 promotes cell proliferation by upregulating human cyclin, glycolytic genes. The promotion of cell migration might be related to *MMP9* upregulation by Six1 directly or indirectly. Moreover, Six1 inhibited the transactivation function of the RELA subunit of NF-κB by regulating the expression of cofactor genes (*AES* and *FUS*) and then suppressing the expression of *MCP-1*. Finally, we confirmed that the overexpression of Six1 in mouse kidney resulted in promoting cell proliferation and migration and suppressing the infiltration of Mophs with consequent anti-inflammation to reduce kidney damage caused by I/R injury (Figure 8).

SIX1 is known to play an essential role during early metanephric kidney development (Kawakami et al., 2000). We observed a close timely correlation between cell proliferation/migration and the re-expression of Six1 in mouse TECs, which may indicate a functional link. After I/R injury, Six1 re-expression was increased at 1 day, and peaked at 2 days, before decreasing at 3 days. This re-expression pattern was similar to its expression during development and in parallel with other nephrogenic gene expression patterns in previous findings (O'Brien et al., 2016; Imgrund et al., 1999). The regulated transient expression of Six1 after I/R might correlate with the regulated and timely-restricted proliferative state of the tubular epithelia. On the other hand, the expression of Six1 also might be compromised by immune cell mediated microenviroment transformation at 3 days. It remains to be shown which factors induce the transient upregulation of Six1 after I/R injury and how Six1 expression is downregulated after its transient expression in I/R injury kidney. In our study, we demonstrated in an *in vitro* hypoxia/reoxygenation cell culture system that human SIX1 upregulation was detected at 4–12 h of reoxygenation before decreasing to a low level at 16 h. We suggest that hypoxia/reoxygenation insult leads to SIX1 reactivation, but the mechanisms by which genes target regulated SIX1 transient expression need further investigation.

SIX1 expression ceases in most adult tissues (Ford et al., 1998); however, increased SIX1 expression has been documented in multiple cancers, and the re-expression of SIX1 was shown to promote cell proliferation and migration (McCoy et al., 2009; Li et al., 2018). Whether reactivated SIX1 uses similar gene regulatory networks to regenerate injured epithelia, akin to the one it uses in cancers, is unclear. In this study, we identified that Six1 expression was positively correlated with Ki67 (a cell proliferation marker), and Mmp9 in the early phase of ischemic acute mouse renal failure. Our *in vitro* study confirmed that Six1 promoted cell proliferation and migration, which was evidenced by the enhanced expression of cyclins, glycolytic genes, and *Mmp9*, suggesting that the re-expression of Six1 in tubular may play a key role in the cell cycle progression *in vivo*.

Previous studies demonstrated that Mophs recruitment by hypoxic TECs was a critical mechanism in promoting tubulointerstitial inflammation during ischemia-related AKI (Baek et al., 2015; Li et al., 2019). In line with those findings, the results of our present study demonstrated that the expression of inflammatory genes (*Tnfα* and *Il-1β*) increased concomitantly with the infiltration of F4/80^+^ Mophs in I/R injured mouse kidney. Interestingly, we found a negative correlation between levels of tubular Six1 activation and the infiltration of Mophs as well as the expression of chemokine Mcp-1. Mcp-1 derived from the hypoxic TECs is thought to be essential for infiltration of Mophs (Matsushima et al., 1989). Collectively, these previous studies and our findings encouraged us to explore the possible role of the re-expression of Six1 in mediating Mophs infiltration-induced inflammation.

The expression of *MCP-1* is mainly controlled at the transcription level by many factors, among which NF-κB is the most important (Rovin et al., 1995; Ren et al., 2005). Our previous laboratory studies found that blocking NF-κB activation not only suppressed the expression of *MCP-1* but also reduced the infiltration of Mophs in I/R rat kidney (Cao et al., 2004). Recently, Liu et al. demonstrated that SIX proteins suppressed NIK (NF-κB-inducing kinase)-mediated immunity by negatively regulating non-canonical NF-κB (Liu et al., 2019). In the present study, we found that mouse Six1 repressed the basal and TNFα-stimulated activities of NF-κB *in vitro*. In addition, mouse Six1 overexpression in human TECs exhibited the depressed transcription of *MCP-1*. In contrast, *Six1* knockout mouse TECs exhibited enhanced the transcription of *Mcp-1* under both quiescent and stimulated conditions. Therefore, we speculate that the re-expression of Six1 in TECs may suppress NF-κB activities to reduce *Mcp-1* expression, thus reducing Mophs infiltration *in vivo*.

It was previously reported that the transcriptional activation of NF-κB required multiple cofactor proteins, such as the corepressor AES and the coactivator FUS. These proteins interacted with NF-κB subunit RELA to affect its transcriptional competence (Tetsuka et al., 2000; Uranishi et al., 2001). Li et al. reported that the re-expression of SIX1 in human cancer cell could suppress *FUS* expression (Li et al., 2018). Our results indicated that mouse Six1 could bind to the promoters of *AES* and *FUS*, might regulated their expression, and then inhibited the transactivation function of RELA by protein-protein interactions in human TECs. However, a previous study reported that SIX proteins exhibited gene-proximal inhibitory activities by bounding the promoter regions neighboring the κB sequence (s) of the inflammatory genes (Liu et al., 2019).

To further understand the role of Six1 *in vivo*, we adapted the adeno-associated viral vector serotype 9 (AAV9) administration method to elevate the expression of Six1 in kidney before I/R injury. As expected, we found that the overexpression Six1 ameliorated renal ischemic injury according to both functional and histologic criteria. A biochemistry analysis showed that serum creatinine and BUN levels decreased. Histologically, we observed that tubular damage was markedly reduced. Consistent with our findings in cell models, cell proliferation and migration were increased by upregulating cyclins, glycolytic genes, and *Mmp9*. Moreover, Six1 prevented the infiltration of Mophs by the repressing expression of *Mcp-1*, thus suppressing tubulointerstitial inflammation.

In summary, our study firstly demonstrated that nephrogenic protein Six1 is reactivated in TECs during the early stage of I/R injury. We discovered an important role of Six1 in promotion of cell proliferation and migration and the suppression of inflammation. Six1 promoted cell proliferation by upregulating cyclins, glycolytic genes and might increase cell migration by upregulating *Mmp9*, and inhibited the infiltration of Mophs by downregulating *Mcp-1*, thus suppressing tubulointerstitial inflammation. These findings provide unique insights into the renoprotective mechanisms of nephrogenic protein and a potential therapeutic target that could be used to ameliorate kidney damage.

## Data Availability

The original contributions presented in the study are included in the article/Supplementary Material, further inquiries can be directed to the corresponding author/s.

## Abbreviations

AKI, acute kidney injury; CKD, chronic kidney disease; I/R, ischemia/reperfusion; SIX1, sine oculis homeobox 1; TECs, tubular epithelial cells; Mophs, monocytes/macrophages; MMP9, matrix metalloproteinase 9; AES, amino-terminal enhancer of split; FUS, fused in sarcoma; NF-κB, nuclear factor-κB; MCP-1, monocyte chemotactic protein-1; BUN, blood urea nitrogen; IRI, ischemia/reperfusioninjury; AAV, adeno-associated viral; H/R, hypoxia/reoxygenation; BrdU, 5-bromodeoxyuridine; HK2, human kidney 2 cell line; TCMK1, mouse renal tubular epithelial cell line; OCT, optimum cutting temperature; ECM, extracellular matrix; BSA, bovine serum albumin; PBS, phosphate buffer saline.
