# Supplementary material for: SIX1 Activation Is Involved in Cell Proliferation, Migration, and Anti-inflammation of Acute Ischemia/Reperfusion Injury in Mice
Source: Front Mol Biosci. 2021 Aug 26;8:725319. doi: 10.3389/fmolb.2021.725319 (PMC8427868; doi:10.3389/fmolb.2021.725319)
Supplement: Supplementary file 1 [file DataSheet1.pdf]

# *Supplementary Material*

## Supplementary Data

## Supplementary Figures and table

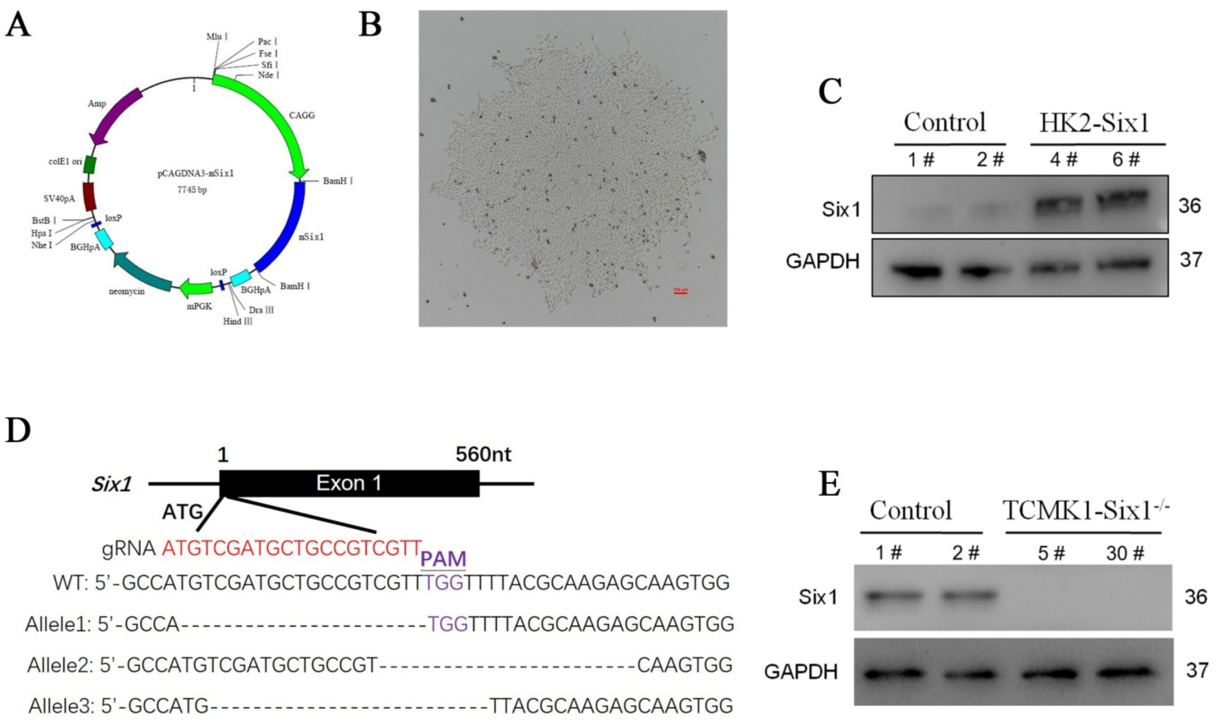


**Supplementary Figure 1.** Mouse Six1 overexpression human proximal tubular epithelial cell lines (HK2-Six1) and mouse renal tubular epithelial Six1 knockout cell lines (TCMK1-Six1$^{-/-}$) were established. **(A)** The structure of the mouse Six1 overexpression vector (pCAGDNA3-mSix1). **(B)** Single clone cell lines were obtained by selection with 200 μg/ml G418 (Gibco) for about 10 days. Bar = 100 μ m. **(C)** Western blotting analysis of Six 1 in the HK2-Six1 cell lines (4# and 6#) and Control cell lines (1# and 2#). **(D)** Confirmation of genetic knockout of Six1 in TCMK1-Six1$^{-/-}$. Schematic representation of Del-base pairing and the sgRNA target locus of exon 1 in Six1. Six1$^{-/-}$ contains deletion of (–) 22 bp, (–) 19 bp, and (–) 20 bp alleles. **(E)** Protein expression of Six1 in the TCMK1-Six1$^{-/-}$ cell lines (5# and 30#) and control cell lines (1# and 2#) determined by Western blotting. GAPDH, glyceraldehydes-3-phosphate dehydrogenase.

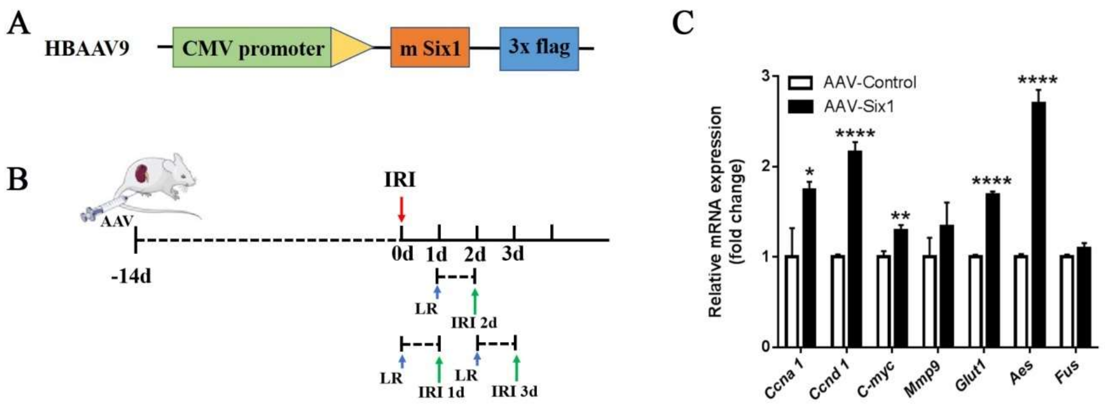


**Supplementary Figure 2.** Six1 overexpress in mice kidney by injecting of the adeno-associated viral vector serotype 9 (AAV9)-Six1. **(A)** The construct of AAV-Six1 vector. **(B)** The strategy for AAV injection and renal IRI. **(C)** mRNA expression of genes (*Ccna 1*, *Ccnd 1*, *C-myc*, *Mmp9*, *Glut1*, *Aes* and *Fus*) in AAV-Six1group, and AAV-Control group before IRI assessed by qRT-PCR. IRI, ischemia/reperfusion injury; LR, Ligation of the right kidney; IRI 1d, 1 day after IRI; IRI 2d, 2 days after IRI; IRI 3d, 3 days after IRI.

**Supplementary Table 1.**The primers used for qRT-PCR

| Gene | Sense/antisense | Fragment |
|---|---|---|
| ***Six1*** (mouse) | 5'-AAGGAGGGAACCTGGAACG-3' | 174bp |
| | 5'-GGTGATTGTGAGGCGAGAA-3' | |
| ***Ccna 1*** (mouse) | 5'-CCACCACCCATGCCCAGTCAGA-3' | 199bp |
| | 5'-CCAACCTCCACCAGCCAGTCCA-3' | |
| ***C-myc*** (mouse) | 5'-GAGATGATGACCGAGTTACTTGGAGG-3' | 191bp |
| | 5'-CTCAGGCTGGTGCTGTCTTTGC-3' | |
| ***Ccnd 1*** (mouse) | 5'-TGTGAGGAGCAGAAGTGCGAAGA-3' | 199bp |
| | 5'-GCCGGATAGAGTTGTCAGTGTAGATG-3' | |
| ***Mmp9*** (mouse) | 5'-CACGGCAACGGAGAAGGCAAAC-3' | 166bp |
| | 5'-CGTCCACTCGGGTAGGGCAGAA-3' | |
| ***Mcp-1*** (mouse) | 5'-CACCTGCTGCTACTCATTCACC-3' | 161bp |
| | 5'-ATGTCTGGACCCATTCCTTCTT-3' | |
| ***Tnfα*** (mouse) | 5'-CGTGGAACTGGCAGAAGAGGCA-3' | 125bp |
| | 5'-GTAGACAGAAGAGCGTGGTGGC-3' | |
| ***Il-1β*** (mouse) | 5'-TTCAAATCTCGCAGCAGCACAT-3' | 196bp |
| | 5'-AGCAGGTTATCATCATCATCCC-3' | |
| ***Glut1*** (mouse) | 5'-ATCCTGTTGCCCTTCTGC-3' | 144bp |
| | 5'-CCGACCCTCTTCTTTCATCT-3' | |
| ***Pgk*** (mouse) | 5'-ACAGAAGGCTGGTGGATT-3' | 114bp |
| | 5'-TTGTCTGCAACTTTAGCG-3' | |
| ***Ldha*** (mouse) | 5'-GCGGTTCCGTTACCTGAT-3' | 186bp |
| | 5'-ACCTCCTTCCACTGCTCC-3' | |
| ***Aes*** (mouse) | 5'-CCCAGGTTCTGCCCTATTTGTC-3' | 111bp |
| | 5'-GCTGCTGTCGGATGATGGAGTT-3' | |
| ***Fus*** (mouse) | 5'-GCAACGAGCTGGAGACTGGAAG-3' | 101bp |
| | 5'-CATCTGGCTTAGGTGCCTTACA-3' | |
| ***SIX1*** (human) | 5'-GCCACCAGTTCTCGCCTCACAA-3' | 127bp |
| | 5'-TTTCGGCGCACCCGATATTTGC-3' | |
| ***RELA*** (human) | 5'-TGCCGAGTGAACCGAAAC-3' | 220bp |
| | 5'-TGGAGACACGCACAGGAGC-3' | |
| ***AES*** (human) | 5'-AAGGCTGAACGGGATTTG-3' | 131bp |
| | 5'-GCTGCTGTCGGATGATAGAG-3' | |
| ***FUS*** (human) | 5'-ATACCCAACAAGCAACCC-3' | 137bp |
| | 5'-GCCATAGCCTGAAGTGTCC-3' | |
| ***MCP-1*** (human) | 5'-CTTCTGTGCCTGCTGCTC-3' | 154bp |
| | 5'-TGCTGCTGGTGATTCTTCT-3' | |
